# Association Between Shift Work and Mental Health and Sleep Outcomes in Working Adults: A Systematic Review

**DOI:** 10.7759/cureus.110089

**Published:** 2026-06-02

**Authors:** Sabah Shakeel Shaikh, Abdullah Kilic, Cynthia Okoro, Mriganka Rai, Ramkumar Rajapandian, Sheraz Hakeem, Lubna Mohammed

**Affiliations:** 1 Diabetes and Endocrinology, California Institute of Behavioral Neurosciences & Psychology, Fairfield, USA; 2 Internal Medicine, Hackensack University Medical Center, Montclair, USA; 3 Family Medicine, Richmond Gabriel University, Arnos Vale, VCT; 4 Internal Medicine, Medical University Sofia, Sofia, BGR; 5 Trauma and Orthopaedics, California Institute of Behavioral Neurosciences & Psychology, Fairfield, USA; 6 Internal Medicine, Aga Khan University, Karachi, PAK; 7 Internal Medicine, Principles and Practice of Clinical Research, Harvard School of Public Health, Boston, USA

**Keywords:** anxiety, burnout, depression, insomnia, mental health, night shift, rotating shift, shift work, sleep disturbance, stress

## Abstract

Shift work is the norm in modern industrialised societies and is associated with disruptions in circadian rhythms as well as adverse mental health and sleep outcomes. However, many studies have examined sleep-related and mental health outcomes independently, limiting understanding of their interrelationship. This systematic review aimed to evaluate the association between shift work exposure and both sleep-related and mental health outcomes among adult workers. A systematic review was conducted using a predefined and structured methodology for study identification, selection, and reporting. A comprehensive search of PubMed, Cochrane CENTRAL, Europe PMC, PsycINFO, and ClinicalTrials.gov was performed for studies published between January 2016 and January 2026. Eligible studies included adult shift-working populations that reported both sleep-related and mental health outcomes. Observational studies and randomized controlled trials were included. Study selection, screening, and quality appraisal were performed using established methodological assessment approaches appropriate to the included study designs.

A total of 386 records were identified, of which 36 duplicates were removed. Following screening and full-text assessment of 80 articles, 11 studies met the inclusion criteria. Included studies were conducted across Europe, North America, Asia, and Australia and involved diverse occupational groups, including healthcare workers, correctional officers, and industrial workers. Across studies, shift work exposure was associated with sleep-related outcomes such as insomnia, poor sleep quality, and shift work sleep disorder, as well as mental health outcomes including depression, anxiety, stress, and fatigue. Overall methodological quality was moderate to high. Shift work exposure is associated with adverse sleep-related and mental health outcomes among adult workers. These findings support the need for integrated occupational health strategies addressing both sleep and psychological well-being in shift-working populations.

## Introduction and background

Shift work is an indispensable component of contemporary industrialised societies, with a substantial proportion of the global workforce engaged in non-standard working schedules. Adaptation to shift work requires realignment of preferred sleep-wake timing (chronotype), and individual differences in this adaptation influence the extent to which shift work affects sleep [1-3].

Circadian misalignment associated with shift work can lead to poor sleep quality and excessive daytime sleepiness, consistent with the clinical entity commonly referred to as shift work sleep disorder. Many shift workers experience persistent difficulty adapting their internal biological clocks to irregular or nocturnal work schedules, placing them at increased risk of chronic sleep disturbance. Employees working irregular hours, extended shifts, or night schedules frequently report impaired sleep quality, which has been associated with increased workplace accidents, reduced job performance and satisfaction, heightened stress levels, and cognitive impairment [4-7].

Sleep-related outcomes among shift workers are also closely linked to adverse mental health outcomes. Individuals experiencing insomnia or shift work sleep disorder commonly report symptoms of anxiety, depression, fatigue, and reduced work productivity. Evidence suggests that unpredictable work schedules and limited control over shift patterns are associated with higher rates of depressive symptoms, whereas adequate and restorative sleep may partially mitigate these effects. Although existing literature demonstrates important associations between shift work, sleep-related, and mental health outcomes, further synthesis is required to clarify these relationships across occupational groups and to better understand the role of occupational stress in mediating these effects [8-10].

Most existing research has examined sleep disturbances and mental health outcomes among shift workers independently, often overlooking their interrelationship and the role of shift work as a shared occupational exposure. Greater integration of these domains is needed to better understand how sleep-related and mental health outcomes interact in shift-working populations and to inform evidence-based occupational health and safety policies. Recent evidence has further highlighted the mental health outcomes associated with shift work, particularly in high-demand occupational settings [8,9]. Emerging evidence has also linked shift work sleep disorder and circadian disruption with impaired mental health outcomes and reduced occupational functioning [11-13].

To address this gap, this systematic review aims to evaluate the association between shift work exposure and sleep-related outcomes, as well as mental health outcomes, among adult workers.

## Review

Methods

Study Design and Reporting Guidelines

This systematic review was conducted in accordance with the Preferred Reporting Items for Systematic Reviews and Meta-Analyses (PRISMA) 2020 statement [14]. The review protocol was not prospectively registered in PROSPERO or Open Science Framework (OSF) before study initiation; however, eligibility criteria, databases, search strategy, outcomes of interest, and quality appraisal methods were defined before screening.

Database and Search Strategy

A literature search was conducted across electronic databases and trial registers, including PubMed, Cochrane Central Register of Controlled Trials (CENTRAL), Europe PMC, PsycINFO, and ClinicalTrials.gov. The search strategy combined Medical Subject Headings (MeSH) terms and free-text keywords related to shift work, sleep disturbance, insomnia, and mental health outcomes. Keywords included “shift work”, “night shift”, “rotating shift”, “sleep disturbance”, “insomnia”, “mental health”, “depression”, “anxiety”, “stress”, and “burnout”. Boolean operators (AND/OR) were used to refine the search strategy. Only studies published in English involving adult human participants between January 2016 and January 2026 were included. The full search strategies for each database are presented in Table 1.

**Table 1:** Articles identified using each database The final database search was conducted in January 2026 using database-specific combinations of controlled vocabulary and free-text keywords with Boolean operators (AND/OR).

Search strategy	Database used	Number of papers identified
(“shift work” OR “night shift” OR “rotating shift”) AND (“sleep disturbance” OR insomnia OR “sleep quality” OR “circadian rhythm” OR “shift work sleep disorder”) AND (“mental health” OR depression OR anxiety OR stress OR burnout)	PubMed	73
(“shift work” OR “night shift” OR “rotating shift”) AND (“mental health” OR depression OR anxiety OR insomnia OR sleep)	Cochrane CENTRAL	115
(“shift work” OR “night shift” OR “rotating shift”) AND (“mental health” OR depression OR anxiety OR stress OR burnout OR insomnia)	ClinicalTrials.gov	112
(“shift work” OR “night shift” OR “rotating shift”) AND (“mental health” OR depression OR anxiety OR stress OR burnout OR insomnia)	PsycINFO (PsycArticles)	11
(“shift work” OR “night shift” OR “rotating shift”) AND (“mental health” OR depression OR anxiety OR stress OR burnout OR insomnia OR “sleep disturbance”)	Europe PMC	75

Inclusion and Exclusion Criteria

Studies were eligible for inclusion if they examined adult shift workers and assessed both sleep-related and mental health outcomes within the same study. The study designs identified were both observational (cross-sectional, cohort, and case-control) and randomised controlled, where sample-based changes in shift work, sleep, and mental health had been observed and studied with reference to data from the study and the literature used. We considered only relevant English papers. Studies were excluded if they focused solely on sleep outcomes or mental health outcomes without assessing both domains concurrently, if shift work was not a primary exposure of interest, or if the population consisted exclusively of non-shift workers. Shift work exposure was defined as employment involving non-standard working hours, including night shifts, rotating shifts, extended shifts, or irregular work schedules. Qualitative studies, conference abstracts, editorials, commentaries, case reports, review articles, animal studies, and studies lacking full-text availability were also excluded.

This criterion was applied because the review specifically aimed to evaluate the interrelationship between sleep-related and mental health outcomes in shift-working populations.

Selection Process

All records identified through database and register searches were imported into Rayyan systematic review software (Rayyan, Cambridge, USA), where duplicate records were identified and removed. Following deduplication, titles/abstracts and full texts were screened by a primary reviewer and verified by a second reviewer. Any uncertainties at this stage were resolved through mutual discussion among the co-authors. The study selection process and reasons for exclusion at each stage are summarized using a PRISMA 2020 flow diagram.

Quality Appraisal and Risk of Bias Assessment

Two reviewers assessed the methodological quality of the included studies. Any discrepancies in scoring were resolved through discussion and consensus. Quality appraisal tools were selected based on the study design. Observational studies, including cohort, longitudinal, and cross-sectional designs, were evaluated using the Newcastle-Ottawa Scale (NOS), adapted for cross-sectional studies, which assesses study quality across three domains: selection of study groups, comparability of groups, and ascertainment of outcomes or exposures [15].

The NOS uses a star-based scoring system with a maximum of 9 stars. Studies scoring 7-9 stars were classified as high quality, while those scoring 5-6 stars were considered moderate quality; only studies of moderate to high methodological quality were included in the final synthesis. This approach was adopted to minimise bias and exclude studies with substantial methodological limitations or incomplete outcome reporting.

The randomized controlled trial included in this review was assessed using the Cochrane Risk of Bias 2 (RoB 2) tool (The Cochrane Collaboration, London, UK), which evaluates bias across five domains: bias arising from the randomization process, deviations from intended interventions, missing outcome data, measurement of outcomes, and selection of the reported result [16]. Overall risk of bias judgments were categorized as low risk, some concerns, or high risk.

Sleep-related outcomes, including sleep quality, insomnia, sleep disturbance, sleep duration, and shift work sleep disorder, were explicitly considered during quality appraisal to ensure relevance to the review objectives. Validated instruments reported across included studies included the Insomnia Severity Index (ISI), Pittsburgh Sleep Quality Index (PSQI), Epworth Sleepiness Scale (ESS), Patient Health Questionnaire (PHQ-9), Generalized Anxiety Disorder scale (GAD-7), and Centre for Epidemiologic Studies Depression Scale (CES-D).

Data Synthesis

A quantitative meta-analysis was not performed because of substantial heterogeneity in study designs, occupational populations, shift work definitions, outcome measures, and statistical reporting. Findings were therefore synthesised narratively, with attention to the direction and consistency of associations across studies.

Results

**Figure 1:**
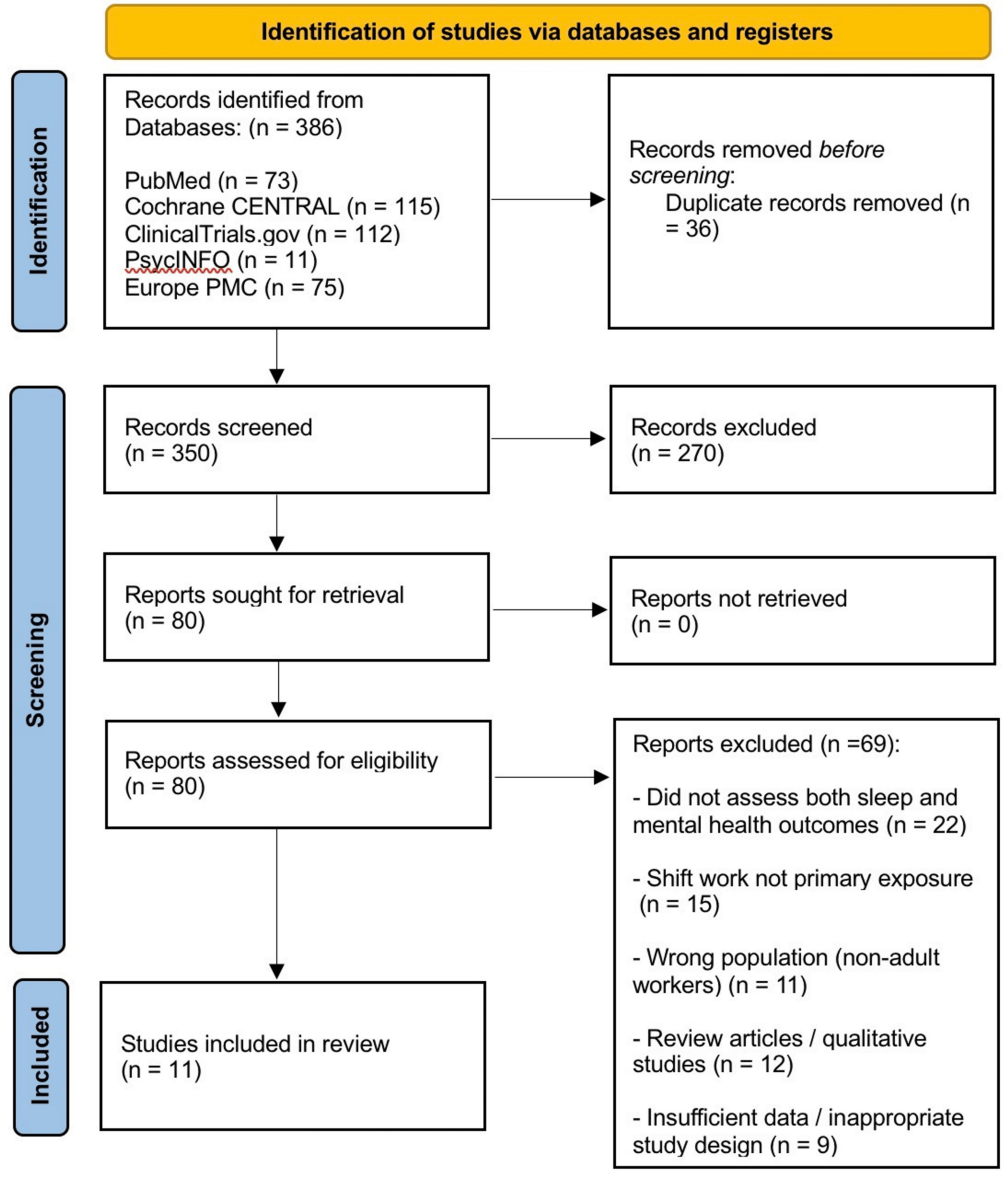
PRISMA 2020 flow diagram of study selection process. PRISMA 2020 flow diagram demonstrating the study selection and screening process for included studies. PRISMA: Preferred Reporting Items for Systematic Reviews and Meta-Analyses

The included studies were conducted across Europe, North America, Asia, and Australia, and involved shift-working populations from healthcare, emergency services, manufacturing, and transportation sectors. Sample sizes ranged from approximately a hundred to several thousand participants. The study selection process is illustrated in the PRISMA 2020 flow diagram (Figure 1) [14].

A total of 386 records were identified through database and register searches. After removing 36 duplicate records, 350 records were screened based on titles and abstracts, of which 270 were excluded. Full-text assessment was conducted for 80 articles, with 69 studies excluded for predefined reasons, including absence of concurrent sleep-related and mental health outcomes, shift work not being the primary exposure, wrong population, review or qualitative study design, or insufficient data. Ultimately, 11 studies met the inclusion criteria and were included in the qualitative synthesis [17-27].

The 11 studies included in this systematic review were conducted across multiple geographic regions, including Europe, North America, Asia, and Australia. The study populations comprised a range of shift-working occupational groups, most commonly healthcare workers (including nurses and hospital staff), emergency service personnel (such as paramedics and correctional workers), and workers in manufacturing and industrial settings.

The majority of included studies employed observational designs, including cross-sectional, cohort, and longitudinal approaches, with one randomized controlled trial. Sample sizes varied substantially across studies, ranging from small occupational cohorts to large population-based samples, reflecting heterogeneity in study design and workforce settings.

Across studies, shift work exposure included night shifts, rotating shifts, extended or irregular schedules, and overtime work. Sleep-related outcomes assessed included sleep quality, insomnia symptoms, excessive daytime sleepiness, and shift work sleep disorder, while mental health outcomes commonly encompassed depression, anxiety, stress, and fatigue.

The studies were conducted across Europe, North America, Asia, and Australia and included diverse occupational groups such as healthcare professionals, paramedics, correctional workers, and industrial workers. The characteristics of the 11 included studies are summarized in Table 2. Outcome measures varied across studies and included validated tools such as the Pittsburgh Sleep Quality Index (PSQI), Insomnia Severity Index (ISI), Patient Health Questionnaire-9 (PHQ-9), CES-D, and occupational stress questionnaires.

**Table 2:** Characteristics of Included Studies (n = 11) RCT: randomised controlled trial; PSG: polysomnography.

Author	Year	Country	Study Design	Sample Size	Occupational Group	Sleep Outcomes	Mental Health Outcomes	Key Findings
Khan et al. [17]	2020	Australia	Observational	15	Paramedics	Sleep restriction, sleepiness	Stress, fatigue	Night shift reduced sleep and increased stress
Vallières et al. [18]	2024	Canada	RCT	43	Healthcare workers	Insomnia severity	Anxiety, depression	Behavioural therapy improved sleep and mental health
Slavish et al. [19]	2022	USA	Longitudinal diary	392	Nurses	Sleep duration, efficiency	Daily stress	Bidirectional stress–sleep association
Vitale et al. [20]	2023	Italy	Cross-sectional	408	Nurses	Insomnia	Anxiety, depression, stress	No significant difference by shift type
Ahn et al. [21]	2024	South Korea	Cross-sectional	6,654	Mixed workers	Insomnia, sleepiness	Depression	Eveningness more common in shift workers
d’Ettorre et al. [22]	2020	Italy	Cross-sectional	300	Nurses	Insomnia, sleepiness	Job stress	Social support reduced insomnia
Reynolds et al. [23]	2022	Australia	Cohort	660	Young workers	PSG-confirmed sleep disorder	Anxiety, depression	Sleep disorder predicted mental health
Ricciardelli et al. [24]	2024	Canada	Cross-sectional	943	Correctional workers	Insomnia, sleep duration	Mental disorder symptoms	Poor sleep strongly associated with distress
Jiang et al. [25]	2025	China	Cross-sectional	614	Nurses	Sleep disturbance	Depression	Sleep disturbance mediated depression
Kang et al. [26]	2021	South Korea	Cross-sectional	14,114	Electronics workers	Insomnia	Depression, suicidal ideation	Shift work increased odds of depression
Yun et al. [27]	2022	South Korea	Cross-sectional	27,554	Paid workers	Sleep disturbance	Depressive symptoms	Quick return strongly associated with depression

Overall, the observational studies demonstrated moderate to high methodological quality, with appropriate participant selection, clearly defined shift work exposure, validated sleep and mental health outcome measures, and reasonable control for confounding variables. The methodological quality of the included observational studies, assessed using the Newcastle-Ottawa Scale, is summarized in Table 3.

**Table 3:** Newcastle–Ottawa Scale (NOS) Quality Appraisal of Observational Studies (n = 10) Selection (maximum 4 stars), Comparability (maximum 2 stars), Outcome/Exposure (maximum 3 stars). Scores of 7-9 were considered high quality, 5-6 moderate quality, and ≤4 low quality.

Reference	Study design	Selection	Comparability	Outcome/Exposure	NOS score	Quality
Khan et al. (2020) [17]	Observational field study	★★★	★	★★★	7/9	High
Slavish et al. (2022) [19]	Observational (daily diary + actigraphy)	★★★★	★	★★★	8/9	High
Vitale et al. (2023) [20]	Cross-sectional (online cohort)	★★★	★	★★	6/9	Moderate
Ahn et al. (2024) [21]	Cross-sectional (large online survey)	★★★	★	★★★	7/9	High
d’Ettorre et al. (2020) [22]	Cross-sectional	★★★	★	★★★	7/9	High
Reynolds et al. (2022) [23]	Cohort/longitudinal (objective sleep/PSG)	★★★★	★★	★★★	9/9	High
Ricciardelli et al. (2024) [24]	Cross-sectional (web-based survey)	★★★★	★	★★★	8/9	High
Jiang et al. (2025) [25]	Cross-sectional	★★★	★★	★★	7/9	High
Kang et al. (2021) [26]	Cross-sectional (workforce survey)	★★★★	★★	★★	8/9	High
Yun et al. (2022) [27]	Cross-sectional (national survey secondary analysis)	★★★★	★★	★★	8/9	High

Risk of bias assessment of the included randomised controlled trial by Vallières et al. [18] using the RoB 2 tool demonstrated an overall low risk of bias, although some concerns were identified in the domains of deviations from intended interventions and missing outcome data.

Discussion

Principal Findings

The findings of this systematic review demonstrate consistent associations between shift work exposure, adverse sleep-related outcomes, and mental health outcomes among adult workers. Sleep disturbances, including insomnia, poor sleep quality, excessive daytime sleepiness, and shift work sleep disorder, were associated with shift work across the included studies [17-27]. Simultaneously, shift-working populations frequently reported symptoms of stress, anxiety, depression, fatigue, and psychological distress [17-20,23-27].

Importantly, studies assessing both sleep-related and mental health outcomes within the same investigation demonstrated that these outcomes commonly co-occur, suggesting that sleep disturbance and mental health outcomes are interconnected rather than independent consequences of shift work exposure [18,23-27]. This integrated pattern contrasts with much of the existing literature, which has traditionally examined sleep-related and mental health outcomes separately.

Interpretation and Possible Mechanisms

Non-standard work schedules, extended working hours, and frequent rotations between day and night shifts can disrupt endogenous circadian rhythms and interfere with regular sleep-wake cycles [1-3]. Circadian misalignment and chronic sleep deprivation may contribute to physiological stress responses and impaired psychological functioning. Insufficient or fragmented sleep has been associated with reduced cognitive performance, impaired emotional regulation, and increased vulnerability to stress, which may in turn contribute to symptoms of anxiety and depression [4,6,10].

In addition, psychosocial occupational factors-such as limited job control, unpredictable schedules, and high workload demands-may further exacerbate sleep disruption and psychological distress [9]. Collectively, these findings suggest that sleep disturbance may act as a mediating pathway linking shift work exposure to adverse mental health outcomes.

**Figure 2:**
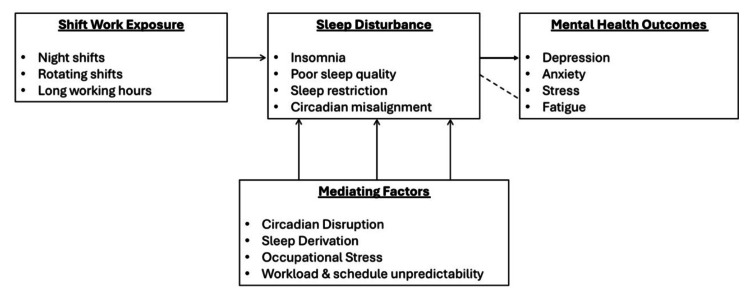
Proposed conceptual framework illustrating the relationship between shift work, sleep-related outcomes, and mental health outcomes based on findings from included studies.

Previous literature has also demonstrated associations between insomnia, depression, suicidality, and circadian disruption among shift-working populations [10-13]. Figure 2 illustrates the distribution and interrelationship of sleep-related and mental health outcomes identified across the included studies, highlighting the multifactorial impact of shift work exposure on worker well-being.

Comparison With Existing Literature

The findings of this review are consistent with previous research demonstrating associations between shift work and sleep disturbance [1,4,5], as well as studies linking shift work to adverse mental health outcomes, particularly depression and psychological distress [8,9]. However, unlike many earlier investigations and reviews that examined either sleep or mental health outcomes separately, this review required the assessment of both outcome domains within the same study. By focusing on shift work as the primary occupational exposure and examining the co-occurrence of sleep and mental health outcomes, this review extends existing occupational health literature and supports the need for integrated conceptual models to understand the health effects of shift work.

Implications for Practice and Policy

From an occupational health perspective, these findings highlight the importance of evaluating both sleep and mental health outcomes when assessing the impact of shift work. Workplace interventions may benefit from addressing shift scheduling practices, ensuring adequate rest periods, and promoting sleep hygiene education [4,7]. Improving worker autonomy and increasing predictability of work schedules may help mitigate adverse psychological outcomes associated with shift work [9]. These findings are particularly relevant for occupational health policy development, workforce planning, and patient and public safety in high-risk occupational settings, where fatigue and impaired mental well-being may have significant consequences.

Strengths

This review was conducted using a PRISMA-guided systematic methodology and incorporated a comprehensive search across multiple databases [14]. By restricting inclusion to studies that assessed both sleep-related and mental health outcomes, this review provides a more integrated understanding of the health effects of shift work. The included studies encompassed diverse occupational groups and geographic regions, enhancing the generalisability of the findings to a broad range of shift-working populations.

Limitations

However, several limitations should be acknowledged. Most included studies were observational, limiting causal inference. There was heterogeneity in outcome measures, occupational settings, and definitions of shift work, which may affect comparability across studies. Only a limited number of randomized controlled trials were identified. Screening and quality appraisal were primarily conducted by a single reviewer, with verification by a second reviewer to minimise the risk of selection bias. Furthermore, publication bias cannot be excluded. The review was not prospectively registered, which may increase the risk of reporting bias. Restricting inclusion to English-language studies may also have introduced language bias. Requiring studies to report both sleep-related and mental health outcomes strengthened the focus of this review but may have excluded studies examining only one outcome domain, limiting generalisability. One included study had a relatively small sample size, which may limit the generalisability of its findings. Most included studies were observational, so causal inference is limited, and reverse causality cannot be excluded.

Future Research

Future research should incorporate longitudinal study designs to better evaluate the temporal relationship between shift work exposure, sleep-related outcomes, and mental health outcomes. Greater standardisation of sleep and mental health outcome measures may facilitate comparisons across studies. Additional interventional studies addressing both sleep-related and mental health outcomes are also encouraged. Further research across different occupational groups, industries, and shift patterns is required to improve understanding of the long-term effects of shift work exposure.

## Conclusions

Shift work is associated with adverse sleep-related and mental health outcomes among adult workers. The findings of this review demonstrate the interconnected nature of these outcomes and support the need for integrated occupational health strategies addressing both sleep and psychological well-being within shift-working populations. These findings have important implications for occupational health practice and policy, particularly in improving shift scheduling and worker wellbeing.
